# Factors Associated with Helmet Therapy Outcomes in Positional Plagiocephaly

**DOI:** 10.3390/jcm15020566

**Published:** 2026-01-10

**Authors:** Sumin Lee, Eunju Na, Joon Won Seo, Seung Ok Nam, Eunyoung Kang, Dong-Hyuk Kim, Sunghoon Lee, Jihong Cheon, Hyeng-Kyu Park, Younkyung Cho

**Affiliations:** 1 Department of Rehabilitation Medicine, Gwangju Christian Hospital, Gwangju 61661, Republic of Korea; leesm681@naver.com (S.L.); naunju@naver.com (E.N.); jwseokorea@gmail.com (J.W.S.); vienna0228@naver.com (S.O.N.); eykang74@hanmail.net (E.K.); north317@daum.net (D.-H.K.); starhoon3@hanmail.net (S.L.); appleisland3@icloud.com (J.C.); 2 Department of Physical and Rehabilitation Medicine, Research Institute of Medical Sciences, Heart Research Center, Chonnam National University Medical School & Hospital, Gwangju 61469, Republic of Korea; phk1118@naver.com

**Keywords:** plagiocephaly, helmet therapy, anterior fontanelle, cranial vault asymmetry

## Abstract

**Background:** Helmet therapy is considered to be a treatment for infants with positional plagiocephaly. Although some studies suggest that anterior fontanelle (AF) size may also affect treatment outcomes, evidence and influence remain unclear. The aim of this study is to assess the impact of anterior fontanelle size on the effectiveness of helmet therapy, with the goal of determining the optimal timing and patient criteria for treatment. **Methods:** We conducted a retrospective study of 94 infants treated with helmet therapy for positional plagiocephaly at Kwangju Christian Hospital between January 2020 and December 2021. Patients were divided into two age groups (≤6 months and >6 months) and three SAF quartiles (≤25%, 25–75%, ≥75%). Parameters reflecting the degree of cranial asymmetry correction, including cranial vault asymmetry (CVA) and cranial vault asymmetry index (CVAI), were recorded at the start and end of treatment. **Results:** Infants aged ≤6 months showed significantly greater improvements in cranial vault asymmetry (CVA) and cranial vault asymmetry index (CVAI) compared to older infants (CVA: 4.57 ± 2.30 mm vs. 7.04 ± 3.85 mm, *p* = 0.003; CVAI: 3.10 ± 1.55% vs. 4.45 ± 2.44%, *p* = 0.011). When analyzed by anterior fontanelle (AF) size quartiles (≤25%, 25–75%, ≥75%), no significant differences in treatment outcomes were observed at the end of therapy for CVA (*p* = 0.88) or CVAI (*p* = 0.91). In infants ≤6 months, SAF quartile analysis also showed no significant differences in CVA (*p* = 0.97) or CVAI (*p* = 0.98) improvements. **Conclusions:** Our findings indicate that anterior fontanelle size is not a predictor of helmet therapy outcomes in positional plagiocephaly. Early initiation of helmet therapy (≤6 months) remains the most critical factor for achieving optimal results.

## 1. Introduction

Positional plagiocephaly is a cranial deformation caused by external pressures, leading to asymmetrical skull growth [1]. This condition can arise from a variety of factors, such as birth trauma, assisted delivery, multiple pregnancies, prematurity, congenital muscular torticollis, and prolonged head positioning in infants [2]. Furthermore, intrauterine position, developmental delay, oligohydramnios, bottle propping and common use of car seats may cause this problem [2,3,4,5,6]. Flattening and deformation of the skull typically occur within the first few months of life, mainly influenced by the infant’s head position [7]. Depending on the infant’s age and the severity of the deformity, treatment options include active repositioning therapy, physiotherapy, or cranial molding orthosis (helmet therapy) [8,9]. Notably, the prevalence of positional plagiocephaly has significantly increased following the ‘Back to Sleep’ campaign in the United States, an initiative designed to prevent sudden infant death syndrome (SIDS) by promoting supine sleeping positions [10]. Furthermore, recent studies have emphasized that cranial conformation and the severity of these deformations are significantly influenced by demographic factors, including ethnicity [11,12].

Helmet therapy has been widely recognized as effective for severe positional plagiocephaly since Clarren et al.’s 1979 report [12,13]. However, a 2014 randomized controlled trial questioned its use as a standard treatment for moderate to severe deformities in healthy infants [8]. In contrast, a 2016 meta-analysis supported helmet therapy for moderate to severe cases, particularly in older infants, despite the lack of standardized criteria for treatment timing and severity [14]. Recently, several studies have provided evidence that helmet therapy can result in equivalent or superior treatment outcomes compared to repositioning, with the added benefit of shorter treatment duration [15,16].

Skull deformation is most commonly observed between two (16–22%) and four months (20%) after birth, and the prevalence drops when infants grow older [2,17]. Many studies indicated that initiating helmet therapy early (between 3 and 5 months of age) results in better outcomes compared to starting therapy after 6 months of age. Additionally, treatments such as physiotherapy and head repositioning are often less effective after the first 6 months of life [18]. These findings highlight the importance of early intervention and suggest the need for clear criteria to guide the timing and initiation of helmet therapy for infants with positional plagiocephaly.

In addition to age, anterior fontanelle (AF) size may be an important factor influencing helmet therapy outcomes [19]. AF serves as an indicator of skull growth from the fetal stage through early infancy [20]. AF typically closes around 12 to 18 months of age, following an initial enlargement in the first two months postnatally, followed by gradual size reduction [21]. This growth pattern suggests that AF size could serve as a potential index of cranial growth, potentially influencing the effectiveness of helmet therapy.

Previous studies have examined whether anterior fontanelle size could serve as an additional parameter for the indication of helmet therapy. Kim et al. reported that infants with larger AF sizes, estimated by transverse diameter, tend to achieve better outcomes with helmet therapy [19]. Conversely, another study has suggested that patients with smaller AF sizes may have reduced cranial remodeling potential, thereby necessitating helmet therapy [22]. However, methods for measuring AF size vary widely between studies, and the clinical reliance on less precise techniques like skull X-rays may limit its predictive value [23]. While studying the cases, we hypothesized that a larger AF size might facilitate greater cranial movement and thus lead to better treatment outcomes.

Given these considerations, this study aims to identify optimal candidates and timing for helmet therapy by examining the relationship between AF size measurements and the effectiveness of helmet therapy. By focusing on AF size, we seek to determine whether it serves as a reliable predictor of treatment outcomes, similar to age, and provide clearer criteria for guiding helmet therapy recommendations.

## 2. Materials and Methods

### 2.1. Data Source

A retrospective analysis was conducted on 103 patients treated with helmet therapy for positional plagiocephaly at the Department of Rehabilitation Medicine in Kwangju Christian Hospital between January 2020 and December 2021.

### 2.2. Study Population

For this study, the dataset included patients diagnosed with positional plagiocephaly who wore helmet orthoses (Gio Inc., Seoul, Republic of Korea) for more than 18 h per day. Age at the start of helmet therapy was recorded as corrected age, accounting for prematurity. Helmet adjustments were performed every four weeks. Patients were selected for helmet therapy based on cranial measurements, with treatment typically recommended for those with a cranial vault asymmetry (CVA) of 10 mm or greater or a cranial vault asymmetry index (CVAI) of 6.25% or higher, indicating moderate to severe plagiocephaly. Baseline and post-treatment data were compared to investigate the relevance of age and anterior fontanelle size on the effectiveness of helmet therapy. Patients who completed helmet therapy for less than 8 weeks or discontinued treatment early were excluded from the analysis. Patients who discontinued treatment before reaching the threshold were included in the analysis if the duration of helmet therapy exceeded 8 weeks. Treatment completion criteria included achieving a CVA reduction to ≤5 mm. However, for patients aged 24 months or older, treatment was considered complete if it was discontinued due to poor compliance at the caregiver’s request. Ultimately, 94 patients met the eligibility criteria and were included in the study. All participants included in this study were of South Korean nationality and East Asian ethnicity.

### 2.3. Measurements

**Figure 1:**
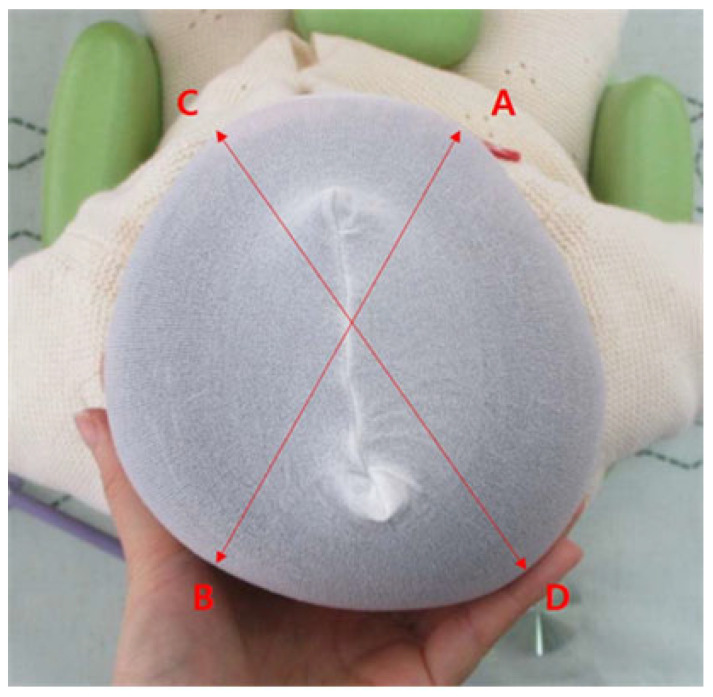
Cranial vault asymmetry (CVA) and CVA index (CVAI) measurements. Left and right frontozygomatic arch (A, C) and the contralateral euryon (B, D) are shown. Abbreviation: CVA, Cranial vault asymmetry; CVAI, Cranial vault asymmetry index. CVA = |AB − CD|(mm), CVAI = |AB − CD|/min [AB, CD] × 100 (absolute diagonal difference/shorter diagonal distance × 100).

Patient characteristics were collected, including sex, age at start and completion of helmet therapy, duration of helmet therapy. Head circumference, cranial vault asymmetry (CVA), cranial vault asymmetry index (CVAI), anterior asymmetry ratio (ASR), posterior asymmetry ratio (PSR), skull volume were measured with the Human body 3D scanner (Creaform Inc., Quebec, QC, Canada). We measured these variables at the start and completion of the helmet therapy. CVA was defined as the absolute difference between two cranial diagonals: AB (right frontozygomatic point to left occipital prominence) and CD (left frontozygomatic point to right occipital prominence) [18,24] (Figure 1).

**Figure 2:**
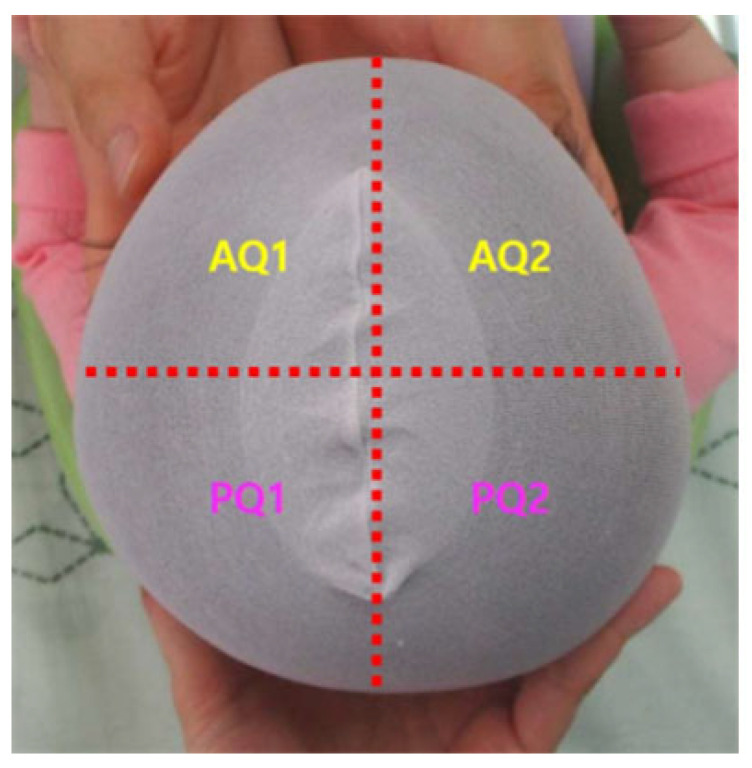
Dotted lines connecting the glabella, tragus and opisthocranion are shown. The two anterior quadrants (AQ) are AQ1 and AQ2. The two posterior quadrants (PQ) are PQ1 and PQ2. Abbreviation: ASR, anterior symmetric ratio; PSR, posterior symmetric ratio. ASR = the smaller of AQ1 and AQ2/the larger one, PSR = the smaller of PQ1 and PQ2/the larger one.

To further evaluate the symmetry and quantify the changes, the skull was mathematically partitioned into four distinct quadrants using anatomical landmarks. The imaginary lines were drawn connecting the glabella, the tragus, and the opisthocranion [19] (Figure 2).

**Figure 3:**
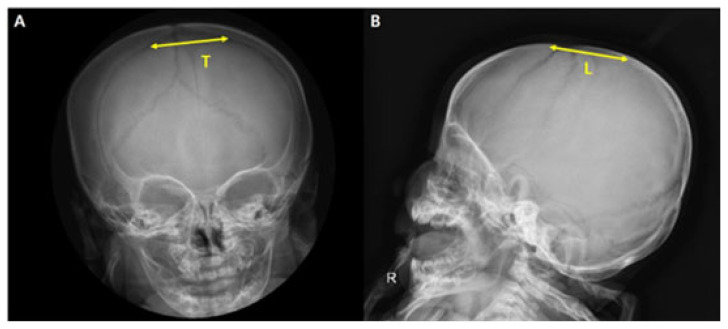
Radiographs of skull in anteroposterior (**A**) and lateral (**B**) views are shown. Abbreviation: T = Transverse diameter, L = Longitudinal diameter. Size of anterior fontanelle (SAF) = T + L/2.

Cranial vault asymmetry index (CVAI) was calculated as the absolute difference between the longer and shorter cranial diagonals (|AB − CD|), divided by the shorter diagonal (AB or CD), and multiplied by 100 [15,25]. We defined the reduction rate as the monthly decrease in the diagonal difference before and after correction, divided by the correction period. The growth rate was defined as the monthly increase in the difference in head circumference before and after correction. The size of anterior fontanelle was measured with the skull anteroposterior (AP) and lateral view x-ray. Transverse diameter(T) of anterior fontanelle was measured in the AP view, and the longitudinal diameter(L) was measured in the lateral view. The average of the two diameters was defined as size of anterior fontanelle (SAF) [26] (Figure 3).

### 2.4. Statistical Analysis

Data analysis was performed using SPSS software version 21 (IBM Corp., Armonk, NY, USA). Descriptive statistics were used to summarize the demographic and clinical characteristics of the study population. To assess changes over time and between groups, Generalized Estimating Equation (GEE) was conducted. GEE was used to evaluate both within-group changes over time (start vs. end) and between-group differences (≤6 months vs. >6 months) in outcome measures, accounting for the repeated nature of the data. The time effect, group effect, and their interaction were tested to determine the significance of changes. Given the significant time effects observed in the GEE analysis, additional non-parametric tests were applied due to the non-normal distribution of the data. For within-group comparisons, the Wilcoxon signed-rank test was used to compare the start and end values of cranial vault asymmetry (CVA) and cranial vault asymmetry index (CVAI). The Mann–Whitney U test was applied to independently compare the start and end values between two age groups. For anterior fontanelle (AF) size comparisons, infants were categorized into three groups based on SAF quartiles (≤25%, 25–75%, ≥75%) for between-group comparisons. Between-group differences in treatment outcomes across SAF groups were evaluated using the Kruskal–Wallis test. For all analyses, a *p*-value less than 0.05 was considered statistically significant.

## 3. Results

In total, 94 infants (48 males and 46 females) with positional plagiocephaly were evaluated. Based on the patient groups by age at the start of helmet therapy, 63 patients were 6 months old or younger, and 31 patients were older than 6 months. Mean values for anterior fontanelle size and head measurements are shown (Table 1).

**Figure 4:**
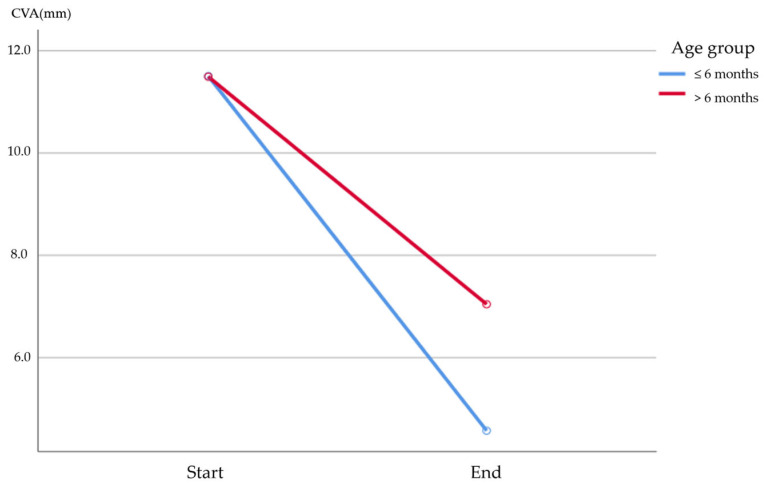
Comparison of Cranial Vault Asymmetry (CVA) Between Age Groups (≤6 months vs. >6 months) Over Time. Abbreviation: CVA, Cranial vault asymmetry.

To assess changes over time and between groups, a Generalized Estimating Equation (GEE) was conducted (Figure 4). GEE was performed to examine both within-group changes over time and between-group differences in the outcome measures.

The results revealed that there was no significant difference between the groups in CVA (*p* = 0.087). However, a significant change over time was observed (*p* < 0.001), indicating that both groups experienced substantial within-group changes in CVA during helmet therapy.

Given the significant time effects observed with GEE, within-group comparisons of the start and end values were performed using the Wilcoxon signed-rank test (Table 2). Both the ≤6 months and >6 months groups showed significant changes over time. Specifically, for both cranial vault asymmetry (CVA) and cranial vault asymmetry index (CVAI), significant reductions were observed from the start to the end within each group (CVA: *p* < 0.001, CVAI: *p* < 0.001).

For between-group comparisons, Mann–Whitney U tests were performed independently on the start and end values. The Mann–Whitney U test was used to assess differences between the groups at the start and end, as the groups were not normally distributed, and it allowed for a comparison of the two independent groups at each time point. At the start, there was no significant difference between the two groups (*p* = 0.907). However, at the end of the study, the two groups exhibited significant differences, with the ≤6 months group showing greater improvement compared to the >6 months group. Specifically, at the end of treatment, the ≤6 months group achieved significantly lower values in CVA (*p* = 0.003) and CVAI (*p* = 0.011).

Table 3 compares treatment outcomes based on anterior fontanelle (AF) size. Infants were divided into three groups: those with AF sizes in the lower quartile (≤25%), the middle quartile (25–75%), and the upper quartile (≥75%).

For CVA, the mean values at the start were 10.99 ± 4.48 mm, 11.27 ± 3.83 mm, and 12.50 ± 4.28 mm for the SAF ≤ 25%, 25% < SAF < 75%, and SAF ≥ 75% groups, respectively (*p* = 0.31). At the end, the mean values were 5.75 ± 3.45 mm, 5.22 ± 3.02 mm, and 5.40 ± 3.08 mm, with no significant differences observed (*p* = 0.88).

For CVAI, the mean values at the start were 6.90 ± 2.98%, 6.92 ± 2.47%, and 7.82 ± 2.72% for the SAF ≤ 25%, 25% < SAF < 75%, and SAF ≥ 75% groups, respectively (*p* = 0.25). At the end, the mean values were 3.78 ± 2.29%, 3.45 ± 1.93%, and 3.55 ± 1.89%, with no significant differences observed (*p* = 0.91).

To evaluate the possibility of age-related bias, Table 4 focused on infants aged ≤6 months, using the same SAF quartiles (≤25%, 25–75%, ≥75%). At the start of helmet therapy, no significant differences in CVA (*p* = 0.47) or CVAI (*p* = 0.34) were observed among the SAF quartiles. Similarly, at the end of therapy, the SAF quartiles did not significantly affect the outcomes, with *p*-values of CVA (*p* = 0.97) or CVAI (*p* = 0.98).

## 4. Discussion

Helmet therapy is commonly used to manage positional plagiocephaly in infants, with studies consistently reporting its effectiveness in improving cranial asymmetry and preventing progression [27]. While a randomized controlled trial targeting 84 infants aged 5 to 6 months reported no additional effect of helmet therapy for facial asymmetry compared to natural development, most studies have reported that helmet therapy is effective in correcting plagiocephaly and mid-facial asymmetries [8,27,28,29,30]. Among these, Kim et al. suggested that larger size of anterior fontanelles (AF) is associated with greater improvements in cranial vault asymmetry (CVA) and cranial vault asymmetry index (CVAI) [19]. Given the potential influence of both age and AF size on treatment outcomes, our study aimed to investigate the optimal candidates and timing for helmet therapy. Specifically, we examined the relationship between AF size and the effectiveness of helmet therapy while also considering the role of age at the start of treatment. Our results indicate that the effectiveness of helmet therapy increases when initiated at an early age (Table 2). Younger infants, who experience faster cranial growth, benefit more from the remodeling effect of helmet therapy. This aligns with previous research suggesting that therapy is most effective when started between 3 and 6 months of age [23,27,31]. This implies that the remodeling effect of helmet therapy may decrease as cortical density increases with continued bone growth. This finding highlights the importance of initiating helmet therapy before 6 months of age to achieve optimal outcomes. Based on our results, we recommend starting helmet therapy as early as possible, ideally before 6 months of age, to maximize its effectiveness. However, our findings also demonstrate that helmet therapy remains effective even beyond 6 months of age, and it should still be considered for older infants (Figure 4). Previous studies have reported that helmet therapy remains effective in older infants, with some research supporting its use up to 18 months of age [18] and others demonstrating significant therapeutic results even when treatment is initiated after 6 months of age [32]. Thus, for infants with positional plagiocephaly under 6 months of age, helmet therapy should be strongly recommended. Even for those older than 6 months, helmet therapy may still be suggested after considering various medical factors.

In our study, anterior fontanelle size did not significantly influence the effectiveness of helmet therapy in correcting positional plagiocephaly. Despite prior studies proposing a potential relationship between larger anterior fontanelles and greater improvements in cranial asymmetry, our analysis revealed no significant differences in treatment outcomes (CVA or CVAI improvements) across SAF quartile groups (≤25%, 25–75%, ≥75%) in both the overall cohort (Table 3) and infants aged ≤6 months (Table 4). These findings indicate that the effect of helmet therapy is not dependent on the anterior fontanelle size. The lack of association between AF size and treatment outcomes might reflect the multifactorial nature of cranial remodeling during helmet therapy. AF size can be influenced by various factors, such as gestational age, mode of delivery and birth weight [33]. The variability in these factors across patient groups may contribute to the lack of a clear relationship between AF size and treatment outcomes. These findings underscore the importance of prioritizing age as the primary determinant for initiating helmet therapy rather than relying on anterior fontanelle size. In other words, our results suggest that helmet therapy can be clinically effective and provide significant improvement in cranial asymmetry even in infants with a smaller SAF. Therefore, a smaller fontanelle size should not be considered a limiting factor or a reason to delay the initiation of cranial molding orthosis.

Furthermore, measurement methods may also play a role. We utilized skull X-rays (AP and lateral views), which are widely used for positional plagiocephaly assessments. Furthermore, measurement methods should be considered in the context of clinical practice. In this study, we utilized skull X-rays (AP and lateral views), which are the standard and most widely implemented diagnostic tool for the initial assessment of positional plagiocephaly. While 3D-CT can provide highly detailed anatomical images, its use is primarily essential for the differential diagnosis of craniosynostosis rather than routine assessment. Although X-ray imaging may offer a different level of granularity compared to 3D-CT or ultrasonography in measuring AF size, it remains a highly practical and reliable method in a real-world clinical setting. Given the need to balance diagnostic detail with concerns such as cost and radiation exposure, the use of X-rays represents a clinically sound approach for evaluating the majority of plagiocephaly cases. Future studies incorporating ultrasonography to measure cranial parameters, such as skull thickness [34], could provide further insights by identifying which imaging modality correlates more strongly with helmet therapy effectiveness.

Additionally, the potential influence of head circumference on the efficacy of helmet therapy deserves careful consideration. While the rate of cranial expansion is thought to be related to the degree of asymmetry correction, defining the independent role of head circumference itself remains challenging. According to Lindemann et al., a significant decrease in head circumference growth was observed following helmet therapy. Since helmet therapy changes the natural growth of the head circumference, it is hard to distinguish whether the improvement comes from the initial head size or the effect of the helmet itself [35]. Therefore, further studies are necessary to clarify the relationship between the head circumference and treatment outcomes.

According to Foster et al. [11], demographic associations play a critical role in the clinical presentation of pediatric cranial deformations. Specifically, while East Asian infants often exhibit a higher baseline cephalic index (CI) compared to other ethnic groups, their research further indicates that parameters for positional plagiocephaly, such as CVA, do not differ significantly across ethnic backgrounds. In our study, CVA was established as the primary outcome to assess changes before and after treatment, while CI was not included as a study variable. Future research considering ethnic factors for both CVA and CI enhancement would be beneficial for a more comprehensive assessment.

There were some limitations in this study. First, we did not compare the effects of helmet therapy combined with physiotherapy, such as repositioning, which is known to influence cranial asymmetry. Future studies should consider these factors to accurately describe the correlation between the effects of helmet therapy and other treatment factors. Second, the anterior fontanelle size was not accurately measured using 3D-CT or ultrasonography [23]. To verify a truly accurate correlation, using three-dimensional measurements such as those obtained from 3D-CT is necessary in further research. Third, the actual duration of helmet therapy was defined as the period from the helmet prescription date to the helmet removal date. But helmet therapy comes with burdens such as cleansing, checking for correct fit, and potential skin irritation, which can significantly affect compliance. Although wearing the helmet for more than 18 h a day was recommended and monthly check-ups were conducted, accurately assessing compliance in an outpatient setting proved challenging. Therefore, future research should consider precise measurements of duration of helmet therapy. Also, secondary outcomes such as parental satisfaction, anxiety levels, motor development, and quality of life in infants are willing to be considered in future research.

## 5. Conclusions

Our study highlights that the timing of helmet therapy initiation is the most critical factor affecting its success, with earlier intervention (before 6 months of age) leading to significantly better outcomes. While helmet therapy remains effective even beyond 6 months, anterior fontanelle size did not significantly influence treatment outcomes in patients with positional plagiocephaly in our study. Based on our findings, we recommend prescribing helmet therapy primarily according to the patient’s age rather than relying on the anterior fontanelle size. Further large-scale studies are needed to confirm these findings.

## Figures and Tables

**Table 1:** Participant Characteristics and Clinical Measurements.

	Total (n = 94)	≤6 Months (n = 63)	>6 Months (n = 31)
Age (Months)	5.83 ± 1.87	4.76 ± 0.93	8.00 ± 1.34
Sex, no			
Male (%)	48 (51.1)	33 (52.4)	15 (48.4)
female (%)	46 (48.9)	30 (47.6)	16 (51.6)
Size of anterior fontanelle (SAF, mm)	39.57 ± 8.70	40.07 ± 8.05	38.55 ± 9.96
Head Measurements ^1^			
CVA (mm)	11.5 ± 4.10	11.50 ± 4.02	11.49 ± 4.31
CVAI (%)	7.13 ± 2.66	7.26 ± 2.64	6.87 ± 2.71
ASR	0.88 ± 0.06	0.88 ± 0.56	0.89 ± 0.05
PSR	0.93 ± 0.04	0.93 ± 0.04	0.92 ± 0.04
Total volume (cm^3^)	831.94 ± 107.44	782.00 ± 84.74	933.43 ± 71.12

^1^ Head measurements were recorded before the initiation of any treatment. Values are presented as mean ± standard deviation or number. Abbreviation: SAF, Size of anterior fontanelle; CVA, cranial vault asymmetry; CVAI, cranial vault asymmetry index; ASR, anterior symmetry ratio; PSR, posterior symmetry ratio.

**Table 2:** Outcome parameters according to age groups: Within-group changes and between-group comparison.

	≤6 Months (n = 63)			>6 Months (n = 31)			Between-Group Comparison ^†^ (≤6 m vs. >6 m)	
	Start	End	*p*-Value	Start	End	*p*-Value	Start *p*-Value	End *p*-Value
CVA (mm)	11.50 ± 4.02	4.57 ± 2.30	<0.001	11.49 ± 4.31	7.04 ± 3.85	<0.001	0.907	0.003
CVAI (%)	7.26 ± 2.64	3.10 ± 1.55	<0.001	6.87 ± 2.71	4.45 ± 2.44	<0.001	0.652	0.011
Reduction rate	0.81 ± 0.43			0.48 ± 0.36			0.03	
Growth rate	4.87 ± 1.60			2.96 ± 1.23			<0.001	
Duration (mo.)	5.51 ± 1.79			6.16 ± 2.83			0.639	

Values are presented as mean ± standard deviation. Within-group changes (Start vs. End) were analyzed using Wilcoxon signed-rank test. Between-group ^†^ comparisons (≤6 m vs. >6 m) were conducted using the two-sample Mann–Whitney U test. A *p*-value < 0.05 was considered statistically significant. Abbreviations: CVA, cranial vault asymmetry; CVAI, cranial vault asymmetry index.

**Table 3:** Outcome parameters according to anterior fontanelle size.

	SAF Group							
	SAF ≤ 25%		25% < SAF < 75%		75% ≤ SAF			
	M	SD	M	SD	M	SD	F	*p*-Value
CVA-Start (mm)	10.99	4.48	11.27	3.83	12.50	4.28	2.343	0.310
CVA-End (mm)	5.75	3.45	5.22	3.02	5.40	3.08	0.261	0.878
CVAI-Start (%)	6.90	2.98	6.92	2.47	7.82	2.72	2.750	0.253
CAVI-End (%)	3.78	2.29	3.45	1.93	3.55	1.89	0.190	0.909

Values are presented as mean ± standard deviation. Between-group comparisons for SAF groups (≤25%, 25–75%, ≥75%) were conducted using the Kruskal–Wallis test. A *p*-value < 0.05 was considered statistically significant. Abbreviations: SAF, Size of anterior fontanelle; CVA, cranial vault asymmetry; CVAI, cranial vault asymmetry index.

**Table 4:** Outcome parameters according to anterior fontanelle size in Aged ≤6 Months.

	SAF Group							
	SAF ≤ 25%		25% < SAF < 75%		75% ≤ SAF			
	M	SD	M	SD	M	SD	F	*p*-Value
CVA-Start (mm)	10.58	5.07	11.29	3.74	12.75	3.49	1.500	0.472
CVA-End (mm)	4.86	3.13	4.49	2.25	4.48	1.59	0.067	0.967
CVAI-Start (%)	6.80	3.45	7.03	2.39	8.15	2.27	2.188	0.335
CAVI-End (%)	3.24	2.14	3.09	1.55	3.03	0.97	0.033	0.984

Values are presented as mean ± standard deviation. Between-group comparisons for SAF groups (≤25%, 25–75%, ≥75%) in infants aged ≤6 months were conducted using the Kruskal–Wallis test. A *p*-value < 0.05 was considered statistically significant. Abbreviations: SAF, Size of anterior fontanelle; CVA, cranial vault asymmetry; CVAI, cranial vault asymmetry index.

## Data Availability

The data supporting the findings of this study are available within this article.
